# Comparative Intestinal Transcriptomics Reveals Sex-Dependent Physiological Signatures in *Mugilogobius chulae* and Supports Sex-Aware Stress Evaluation

**DOI:** 10.3390/ani16142126

**Published:** 2026-07-09

**Authors:** Ning Zhang, Haiming Chen, Jiahao Gao, Yuhan Zhou, Qiwen Jiang, Jianjun Li, Jian Liao, Yanping Zhang, Zhongduo Wang, Yusong Guo, Zhongdian Dong

**Affiliations:** 1 Key Laboratory of Aquaculture in the South China Sea for Aquatic Economic Animal of Guangdong Higher Education Institutes, Fisheries College, Guangdong Ocean University, Zhanjiang 524088, China; zhangn@gdou.edu.cn (N.Z.); chenhaiming11@stu.gdou.edu.cn (H.C.); jhgao2001@163.com (J.G.); zyh134@stu.gdou.edu.cn (Y.Z.); jqw109@stu.gdou.edu.cn (Q.J.); liaojian@gdou.edu.cn (J.L.); ypzhang@gdou.edu.cn (Y.Z.); wangzd@gdou.edu.cn (Z.W.); ysguo@gdou.edu.cn (Y.G.); 2 Guangdong Provincial Key Laboratory of Aquatic Animal Disease Control and Healthy Culture, Zhanjiang 524088, China; 3 Guangdong Provincial Biotechnology Research Institute, Guangdong Provincial Laboratory Animals Monitoring Center, Guangzhou 510663, China; ljj@gdlami.com; 4 Key Laboratory of Marine Ecology and Aquaculture Environment of Zhanjiang, Zhanjiang 524088, China

**Keywords:** *Mugilogobius chulae*, intestinal physiology, sexual dimorphism, RNA-Seq, environmental stress, aquatic animal, sulfamethoxazole

## Abstract

Sex is an important biological variable in animal physiology and environmental-stress research, but its influence on fish intestinal transcriptomic profiles remains insufficiently characterized. In this study, adult female and male *Mugilogobius chulae* were used to characterize baseline intestinal sex differences and to evaluate their relevance to short-term sulfamethoxazole exposure. Fish were assigned to solvent control or nominal 1 mg/L sulfamethoxazole exposure for 7 days, and intestinal tissues were analyzed by histology, RNA sequencing (RNA-Seq), functional enrichment analysis, gene set enrichment analysis, and quantitative real-time polymerase chain reaction validation. Representative histological sections showed qualitative sex-related differences in intestinal morphology. Transcriptomic analyses revealed that intestinal variation was more strongly associated with sex than with pooled treatment status. The pooled female-versus-male comparison identified 85 differentially expressed genes, whereas the pooled treatment-versus-control comparison yielded no significant differentially expressed genes under the current threshold. Functional enrichment and gene set enrichment analyses showed that female-biased intestinal profiles were mainly associated with nutrient absorption, lipid and sterol handling, membrane transport and epithelial interface-related pathways, whereas male-biased profiles were more closely related to RNA processing, oxidative metabolism, translation, protein targeting and mucosal immune signaling. These findings demonstrate sex-dependent intestinal transcriptomic signatures in *M. chulae* and support sex-aware intestinal transcriptomic analysis in aquatic animal omics and environmental-stress evaluation.

## Simple Summary

The fish intestine is important for digestion, immunity, metabolism and overall health. However, many studies of fish intestinal function do not analyze males and females separately. In this study, we compared gene expression in the intestines of adult female and male *Mugilogobius chulae*, a small estuarine goby used in marine fish research. We also included a short exposure to a nominal concentration of sulfamethoxazole, an antibiotic commonly detected in aquatic environments, to examine whether sex could influence how exposure results are interpreted. The results showed clear differences between female and male intestines, and these differences were greater than the overall effect of short-term sulfamethoxazole exposure. Female intestines were more closely associated with nutrient absorption and lipid processing, whereas male intestines showed stronger signals related to gene regulation, energy metabolism and immune functions. These findings show that the fish intestine is not a sex-neutral tissue. Future omics-based studies on aquatic animals should consider sex when analyzing intestinal physiology, health and environmental stress responses, and these results support sex-aware intestinal transcriptomic analysis in aquatic animal omics research.

## 1. Introduction

The intestine is a multifunctional organ in fish, integrating nutrient digestion and absorption with mucosal immunity, endocrine signaling, and metabolic regulation [1]. In aquatic animals, intestinal function is closely associated with nutrient utilization, stress adaptation, disease resistance, and overall health. Fish intestinal homeostasis is closely linked to microbiota-associated gut–brain communication and gut-related metabolic regulatory pathways, making the intestine a central interface between environmental conditions and host physiology [2,3,4,5]. Waterborne contaminants and other environmental stressors can disrupt intestinal epithelial integrity, alter microbial community structure, disturb immune balance, and interfere with metabolic homeostasis in fish [6,7,8,9]. Accordingly, the intestine has become an important target organ for evaluating physiological status, health-related responses, and environmental stress effects in aquatic animals. Transcriptomic analysis provides a genome-wide approach for characterizing intestinal molecular states and identifying biological pathways associated with host physiology and stress responses.

Sex is another fundamental biological variable that may shape intestinal physiology, yet it has often been underappreciated in fish intestinal studies. Although sexual dimorphism is most frequently discussed in gonads, studies in both fish and mammals show that the intestine is not a sex-neutral tissue [10,11,12,13,14,15,16]. In fish, sex-related differences have been reported in intestinal microbial diversity, community structure, and ecological interactions. For example, significant sex differences in Shannon diversity, phylogenetic diversity, and microbial network structure were observed in yellow drum (*Nibea albiflora*), whereas sex and body weight jointly influenced intestinal microbial diversity in *Gymnocypris chilianensis* [12,13]. An integrated transcriptomic and metabolomic analysis in common carp further revealed clear sex-biased intestinal molecular profiles, with the female gut enriched for growth-related pathways such as glycolysis/gluconeogenesis and riboflavin metabolism, and the male gut enriched for immune- and disease-resistance-associated pathways such as Th17 cell differentiation and autophagy [14]. Sex-specific disease resistance and gut microbiota dynamics have also been observed in juvenile common carp following viral infection [16], and intestinal microbiota shifts markedly during sex reversal in swamp eels (*Monopterus albus*) [15]. In mammals, sex-biased gene expression has been detected in the small intestine and colon, and colonic transcriptomic responses to inflammatory challenges differ between males and females [10,11].

These sex-associated intestinal differences have important implications for omics-based studies of aquatic animal physiology, health, and environmental stress responses. If the intestine exhibits marked baseline sexual dimorphism, sex may act as a major source of biological variation and potentially affect the interpretation of exposure- or stress-induced responses. This concern is supported by exposure studies showing sex-dependent intestinal responses under different environmental or chemical exposure contexts. In fathead minnows, short-term aqueous benzo[a]pyrene exposure shifted gut microbial composition in females rather than males [17]. Diet–gut microbiota associations are also sex-dependent in fish and other vertebrates, indicating that sex can influence not only baseline gut traits but also host responses to environmental factors [18]. However, sex effects are not necessarily uniform across species or exposure contexts. In zebrafish, no obvious sex-specific baseline intestinal microbial pattern was detected, but endocrine-disrupting chemicals such as estradiol and bisphenol A altered intestinal microbiota composition [19]. Sulfamethoxazole (SMX) exposure can induce intestinal microbiota alteration and metabolic disturbance in zebrafish [20], and environmentally relevant levels of SMX can alter the gastrointestinal microbiome of adult male and female zebrafish [21]. Broader biological effects of SMX in adult zebrafish have also been reported [22]. Together, these studies indicate that sex effects in the fish intestine are real but species- and context-dependent and thus should be explicitly considered in intestinal omics and environmental-stress research rather than assumed absent.

*Mugilogobius chulae* is a small estuarine goby widely distributed along the western Pacific coast and is emerging as a useful marine fish model for physiological, genetic, and environmental-stress research. Its small body size, early sexual maturation, high fecundity, frequent spawning, year-round reproduction, ease of laboratory rearing, and available genomic resources and molecular tools provide a solid foundation for omics-based studies in aquatic animals [23,24,25]. This species has already been applied in contaminant-related assessments involving heavy metals, pharmaceuticals, benzo[a]pyrene, and genotoxicity [26,27,28,29,30]. In this study, we compared the intestinal transcriptomes of adult female and male *M. chulae* to characterize baseline sex-biased expression patterns and to evaluate their relevance to the interpretation of short-term SMX exposure. The study aims to clarify whether sex should be incorporated as an experimental factor in fish intestinal transcriptomics and to provide a sex-aware framework for omics-based evaluation of intestinal physiology, aquatic animal health, and environmental stress responses.

## 2. Materials and Methods

### 2.1. Ethics Statement

All experimental protocols were approved by the Animal Research and Ethics Committee of Guangdong Ocean University (Approval No. 202406012). All procedures were conducted in accordance with institutional guidelines for the care and use of experimental animals. The study did not involve endangered or protected species.

### 2.2. Test Chemicals and Experimental Fish

SMX (CAS 723-46-6; purity ≥ 98%) was purchased from Sangon Biotech Ltd. (Shanghai, China). Stock solutions of SMX (10 mg/mL) were prepared in dimethyl sulfoxide (DMSO) and stored at −20 °C in the dark. Adult *M. chulae* were provided by the Guangdong Provincial Biotechnology Research Institute (Guangzhou, China) and maintained at the Key Laboratory of Aquaculture in the South China Sea for Aquatic Economic Animals of Guangdong Higher Education Institutes, Guangdong Ocean University. The fish were reared in artificial seawater at 15 ppt salinity under a 14 h light:10 h dark photoperiod and fed twice daily with newly hatched brine shrimp nauplii. The water temperature was maintained at 26 ± 1 °C. Before exposure, fish were acclimated to laboratory conditions for two weeks. Water quality parameters were monitored regularly during the acclimation and exposure periods, and pH (7.9 ± 0.4) and dissolved oxygen (DO; 6.9 ± 0.4 mg/L) remained stable throughout the experiment.

### 2.3. Experimental Design

Sixteen female (body length = 3.37 ± 0.20 cm, body weight = 0.92 ± 0.20 g) and sixteen male (body length = 3.93 ± 0.30 cm, body weight = 1.54 ± 0.30 g) *M. chulae* aged 18 months were randomly assigned to either a solvent control group or an SMX exposure group, with four replicate tanks per group. Each replicate consisted of a glass tank containing 4 L of artificial seawater and four fish (two females and two males). Fish in the SMX group were exposed to a nominal concentration of 1 mg/L SMX, whereas fish in the solvent control group were maintained in water containing the same concentration of vehicle only. The final concentration of DMSO was 0.1‰ in both the control and SMX treatments. During exposure, fish were maintained under the same temperature, photoperiod and feeding conditions described above. The exposure lasted for 7 days, and 80% of the exposure solution was renewed every 24 h with freshly prepared solution for the corresponding treatment. At the end of the exposure, intestinal tissues were collected after dissection. From each tank, one female and one male were used for histological observation, with the mid-intestinal segment from each fish being fixed in 4% paraformaldehyde (PFA). The whole intestine from the remaining one female and one male was preserved in RNA stabilization solution for total RNA extraction and sequencing. Thus, four biological replicates were obtained for each sex-by-treatment group for RNA sequencing (RNA-Seq).

### 2.4. Histological Analysis

The intestines were fixed in 4% PFA for 24 h and then transferred to 70% ethanol for an additional 24 h. The tissues were dehydrated, paraffin-embedded, sectioned at 4–6 μm thickness, and stained with hematoxylin and eosin (HE) according to a previously described method [31]. Sections were photographed using a Nikon Eclipse Ni-E microscope (Tokyo, Japan).

### 2.5. Total RNA Isolation and RNA-Seq

Total RNA was extracted from intestinal tissues using AG RNAex Pro reagent (Accurate Biotechnology (Hunan) Co., Ltd., Changsha, China) according to the manufacturer’s instructions. Genomic DNA was removed using DNase I (TaKaRa, Dalian, China). RNA quality and concentration were assessed by 1% agarose gel electrophoresis (Sangon Biotech Ltd., Shanghai, China) and a Qubit^®^ RNA Assay Kit with a Qubit^®^ 2.0 Fluorometer (Life Technologies, Carlsbad, CA, USA). All RNA samples met the quality requirements for library construction.

A total of 16 cDNA libraries were constructed, including four female and four male intestinal libraries from the control group and four female and four male intestinal libraries from the SMX exposure group. Library preparation and sequencing were performed by Denovo Biotechnology Co. (Guangzhou, China). All raw sequencing data were deposited in the China National GeneBank DataBase (CNGBdb) under accession number CNP0009254.

Raw reads were filtered by removing reads containing adapters, reads with >10% unknown nucleotides (N), and reads with >50% low-quality bases (Q ≤ 10). Clean reads were aligned to the previously reported *M. chulae* reference genome [24] using HISAT2 v2.1.0 [32]. Transcript assembly was performed using StringTie v1.3.4d [33], and transcript abundance was estimated as FPKM (fragments per kilobase of transcript per million mapped reads). Differential expression analysis was conducted using the R package DESeq2 v1.20.0 based on the raw count matrix [34]. Genes with |log2FC| ≥ 1 and false discovery rate (FDR) < 0.05 were defined as differentially expressed genes (DEGs). Functional enrichment analyses of DEGs were performed based on Gene Ontology (GO) and Kyoto Encyclopedia of Genes and Genomes (KEGG) annotations [35,36].

### 2.6. Principal Coordinate Analysis, PERMANOVA, and Gene Set Enrichment Analysis

Genes with FPKM values greater than 1 in at least 20% of the samples were retained. The filtered FPKM matrix was then used to assess overall transcriptomic relationships among samples by principal coordinate analysis (PCoA). Sample-to-sample dissimilarity was calculated using Euclidean distance. Group differences in global transcriptomic structure were further evaluated by permutational multivariate analysis of variance (PERMANOVA) with 999 permutations [37]. For pooled sex comparison, samples were grouped as F (NCF + TF) and M (NCM + TM), whereas for pooled treatment comparison, samples were grouped as N (NCF + NCM) and T (TF + TM).

To further characterize pathway-level transcriptomic divergence beyond threshold-dependent DEG analysis, gene set enrichment analysis (GSEA) was conducted using ranked gene lists derived from the DESeq2 differential expression results [38]. GO-GSEA and KEGG-GSEA were performed separately for the pooled F vs. M comparison and for the NCF vs. NCM and TF vs. TM comparisons. Genes were ranked according to log2FC values derived from the DESeq2 differential expression results. Enrichment results were interpreted according to enrichment score direction, nominal *p* value, and FDR q value. Gene sets or pathways with a nominal *p* value < 0.05 and FDR q value < 0.25 were considered significantly enriched in the GSEA results. Representative gene sets or pathways were selected for visualization based on statistical significance and biological relevance.

### 2.7. Quantitative Real-Time Polymerase Chain Reaction (qPCR)

Sixteen DEGs (Table S1) were selected for quantitative real-time PCR (qPCR) validation. Total RNA was reverse-transcribed into cDNA using TransScript^®^ All-in-One First-Strand cDNA Synthesis SuperMix (TransGen Biotech, Beijing, China) according to the manufacturer’s instructions. β-actin and *gapdh* were used as internal reference genes, following our previous experimental practice [29]. qPCR was performed using SYBR Green qPCR Mix (GDSBio, Guangzhou, China) on a LightCycler 96 Real-Time PCR System (Roche, Switzerland) according to the manufacturer’s instructions. The qPCR cycling program was as follows: 94 °C for 30 s, followed by 40 cycles of 94 °C for 5 s, 60 °C for 15 s, and 72 °C for 10 s. Melting-curve analysis was subsequently performed at 95 °C for 10 s, 65 °C for 1 min, and 95 °C for 1 s to confirm amplification specificity. Each sample was analyzed with three technical replicates. Relative mRNA expression levels were calculated using the 2^−ΔΔCt^ method [39]. All primer sequences used for qPCR are listed in Table S1.

## 3. Results

### 3.1. Representative Intestinal Histological Features

**Figure 1:**
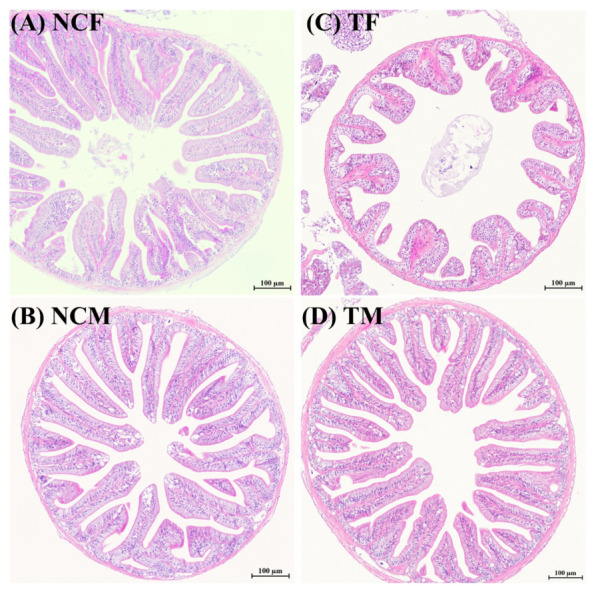
Representative intestinal histology of female and male *M. chulae* under control and nominal SMX exposure. Note: NCF, control female intestine; NCM, control male intestine; TF, SMX-exposed female intestine; TM, SMX-exposed male intestine.

The HE sections showed that the overall intestinal architecture was preserved in all groups. Four intestinal samples were examined for each sex-by-treatment group, and the general histological pattern was consistent within each group. In the control groups, female intestines (Figure 1A) exhibited relatively denser mucosal folds, whereas male intestines (Figure 1B) displayed slenderer and radially arranged folds. After SMX exposure, TF sections showed a relatively enlarged lumen and shorter, blunter mucosal folds (Figure 1C), while TM sections retained a more regular radial organization than TF (Figure 1D). These observations provided a morphological context for the subsequent transcriptomic analyses and suggested qualitative differences in intestinal appearance between females and males under both control and SMX-exposed conditions (Figure 1).

### 3.2. Overall Transcriptomic Variation and DEG Distribution

The electrophoresis profiles indicated that total RNA extracted from all 16 intestinal samples was of sufficient quality for library construction (Figure S1). Sequencing quality was consistently high across libraries, with Q20 values above 97%, Q30 values above 92%, and total mapping rates generally ranging from approximately 79.7% to 83.4% (Table S2).

**Figure 2:**
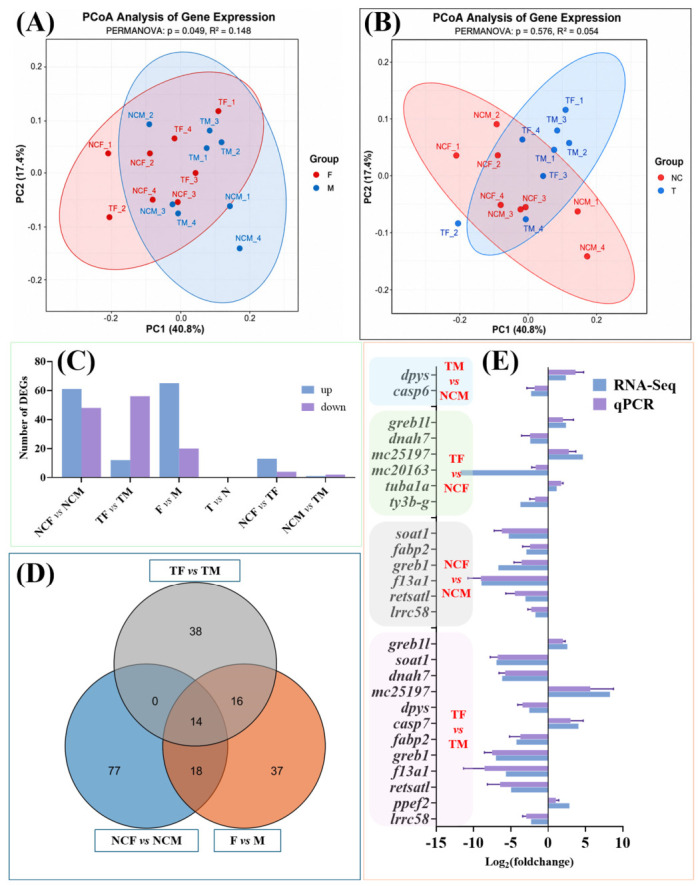
Overall transcriptomic variation, DEG distribution, and qPCR validation in intestinal samples of *M. chulae.* (**A**) PCoA of pooled F vs. M. F = NCF + TF; M = NCM + TM. (**B**) PCoA of pooled N vs. T. N = NCF + NCM; T = TF + TM. (**C**) Numbers of DEGs in pairwise and pooled comparisons. Volcano plots for additional pairwise comparisons are provided in Figure S2. (**D**) Venn diagram of shared DEGs among NCF vs. NCM, TF vs. TM, and pooled F vs. M. (**E**) qPCR validation of representative DEGs.

PCoA analysis showed that intestinal transcriptomic variation was more strongly associated with sex than with pooled treatment status. In the pooled female vs. male comparison, samples showed partial separation between sexes, and PERMANOVA indicated a significant sex effect on overall expression variation (*p* = 0.049, R^2^ = 0.148; Figure 2A). In contrast, the pooled treatment vs. control comparison showed weaker separation, and the PERMANOVA result suggested a limited treatment effect at the global transcriptomic level (*p* = 0.576, R^2^ = 0.054; Figure 2B).

Consistent with this pattern, differential expression analysis showed that sex-related comparisons generated larger DEG sets than the pooled treatment comparison. Specifically, NCF vs. NCM, TF vs. TM, and pooled F vs. M yielded 109, 68, and 85 DEGs, respectively, whereas pooled T vs. N showed no significant DEGs under the current threshold. The within-sex treatment comparisons produced fewer DEGs, with 17 DEGs in NCF vs. TF and only 3 DEGs in NCM vs. TM (Figure 2C; Tables S3–S8). Volcano plots for the additional pairwise comparisons are provided in Figure S2. Venn analysis further identified 14 shared DEGs among NCF vs. NCM, TF vs. TM, and pooled F vs. M, together with comparison-specific DEG subsets (Figure 2D). To validate the RNA-Seq results, 16 selected genes from different comparison groups were examined by qPCR. The qPCR results were generally consistent with the RNA-Seq expression trends (Figure 2E; Table S1).

### 3.3. Sex-Biased DEGs in the Pooled Female vs. Male Comparison

**Figure 3:**
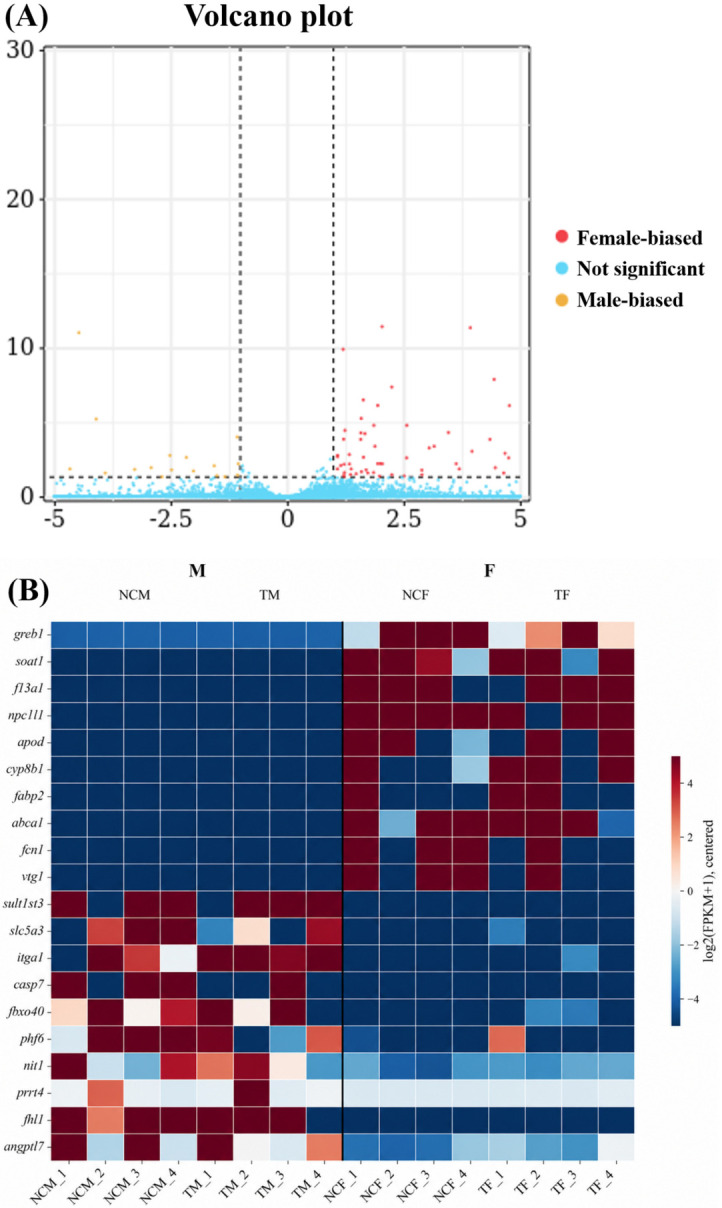
Sex-biased DEGs in the pooled female vs. male intestinal comparison. (**A**) Volcano plot of DEGs in pooled F vs. M. Positive log2FC indicates female-biased expression, whereas negative log2FC indicates male-biased expression. (**B**) Heatmap of representative sex-biased DEGs in pooled F vs. M.

The pooled F vs. M comparison identified sex-biased transcriptional differences in the adult intestine of *M. chulae*. The volcano plot showed significantly upregulated and downregulated genes between pooled females and pooled males (Figure 3A; Table S3). The heatmap of representative DEGs further illustrated this divergence and showed consistent sex-biased expression patterns across the pooled comparison. Female-biased genes included *greb1*, *soat1*, *f13a1*, *npc1l1*, *apod*, *fabp2*, *abca1*, *fcn1*, and *vtg1*, whereas male-biased genes included *sult1st3*, *slc5a3*, *itga1*, *casp7*, *fbxo40*, *phf6*, *nit1*, and *angptl7* (Figure 3B; Table S3). These representative DEGs included genes annotated to lipid or sterol transport/metabolism, solute transport, extracellular or epithelial-related processes, and cell turnover-related categories.

### 3.4. GO and KEGG Enrichment of Pooled Sex-Biased DEGs

**Figure 4:**
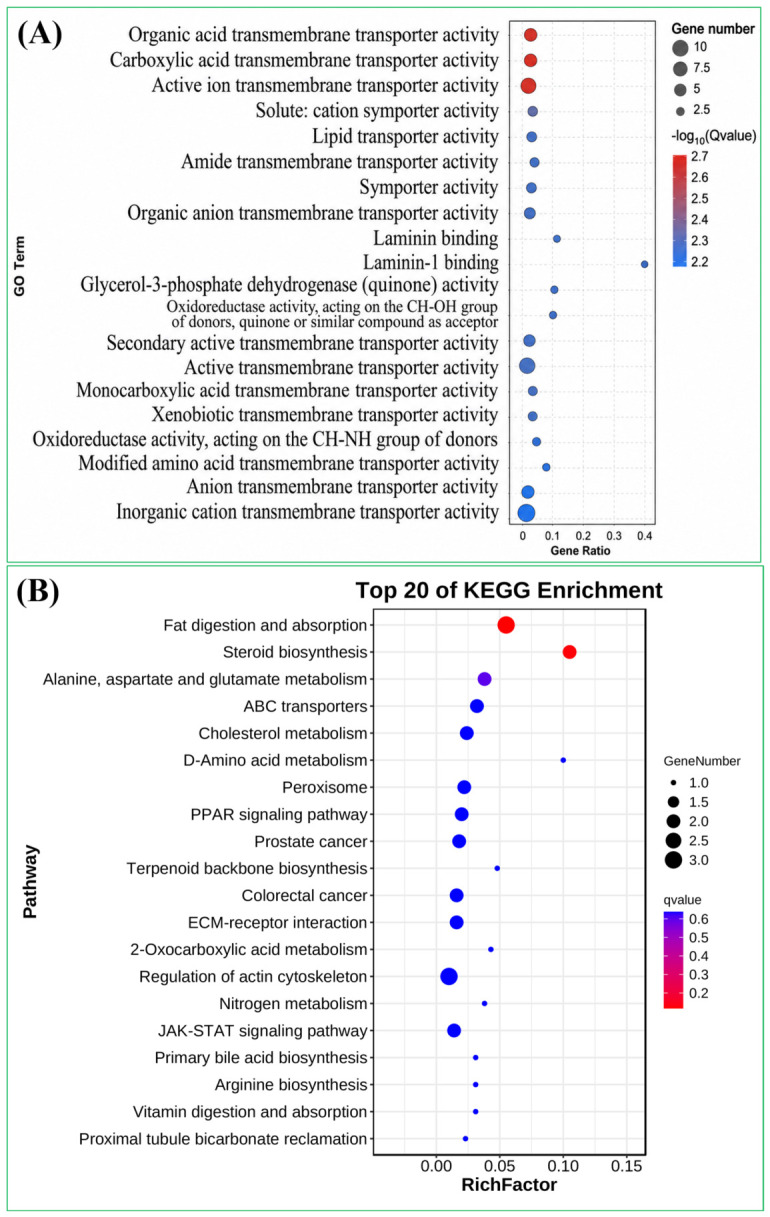
GO and KEGG enrichment analyses of pooled sex-biased intestinal DEGs. (**A**) GO enrichment of pooled F vs. M DEGs. (**B**) KEGG enrichment of pooled F vs. M DEGs.

GO enrichment analysis showed that the pooled sex-biased DEGs were mainly associated with transmembrane transport and membrane-related functions, including organic acid transmembrane transporter activity, carboxylic acid transmembrane transporter activity, active ion transmembrane transporter activity, lipid transporter activity, lipid transport, and intrinsic component of membrane (Figure 4A; Table S9). KEGG enrichment analysis identified pathways related to intestinal physiological functions, including fat digestion and absorption, steroid biosynthesis, ABC transporters, cholesterol metabolism, peroxisomes, and the PPAR signaling pathway (Figure 4B; Table S10). Overall, the enriched GO terms and KEGG pathways were mainly related to nutrient absorption, lipid and sterol handling, membrane transport, and epithelial interface-associated processes.

### 3.5. GO-GSEA Profiles of the Pooled Female vs. Male Comparison

**Figure 5:**
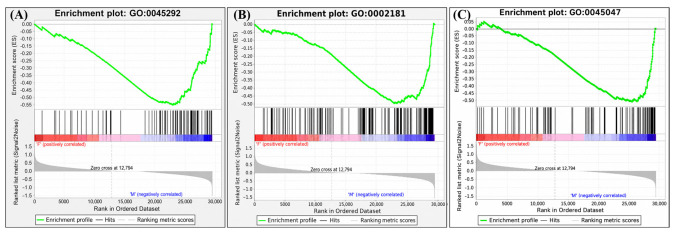
Representative GO-GSEA plots for the pooled female vs. male intestinal comparison. The three representative GO-GSEA plots correspond to: (**A**) GO:0045292 mRNA cis splicing, via spliceosome; (**B**) GO:0002181 cytoplasmic translation; (**C**) GO:0045047 protein targeting to the endoplasmic reticulum.

GO-based GSEA of the pooled F vs. M comparison identified representative gene sets related to mRNA cis splicing via the spliceosome, cytoplasmic translation, and protein targeting the endoplasmic reticulum (Figure 5). Additional representative GO-GSEA plots showed enrichment patterns for the U2-type spliceosomal complex, mRNA cleavage and polyadenylation specificity factor complex, SRP-dependent cotranslational protein targeting to the membrane, and establishment of protein localization to the endoplasmic reticulum (Figure S3). The full GO-GSEA results for the pooled F vs. M comparison are provided in Table S15, including enrichment scores, normalized enrichment scores, nominal *p* values, FDR q values, and leading-edge genes. These GSEA results showed pathway-level differences involving RNA processing and protein handling (Figure 5 and Figure S3).

### 3.6. KEGG-GSEA Profiles of the Pooled Female vs. Male Comparison

**Figure 6:**
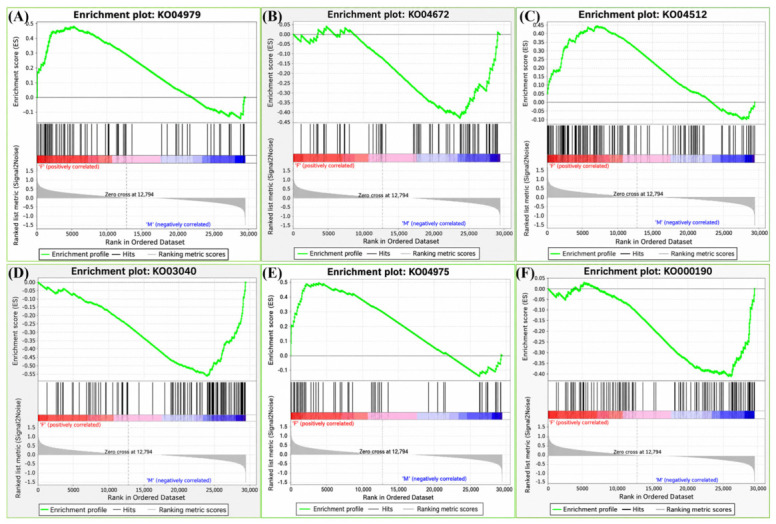
Representative KEGG-GSEA plots for the pooled female vs. male intestinal comparison. (**A**) Fat digestion and absorption; (**B**) Cholesterol metabolism; (**C**) ECM–receptor interaction; (**D**) Spliceosome; (**E**) Oxidative phosphorylation; (**F**) Intestinal immune network for IgA production.

Pathways including fat digestion and absorption, cholesterol metabolism, and ECM–receptor interaction were enriched toward the pooled female side, whereas spliceosome, oxidative phosphorylation, and intestinal immune network for IgA production were enriched toward the pooled male side (Figure 6). Additional representative KEGG-GSEA plots included steroid biosynthesis, ABC transporters, focal adhesion, TGF-beta signaling pathway, ribosome, peroxisome, and other pathways (Figure S4). The full KEGG-GSEA results for the pooled F vs. M comparison are provided in Table S16, including enrichment scores, normalized enrichment scores, nominal *p* values, FDR q values, and leading-edge genes. Together with the DEG and ORA results, the KEGG-GSEA results showed coordinated pathway-level differences between pooled females and pooled males (Figure 6 and Figure S4).

### 3.7. Functional Profiles of NCF vs. NCM and TF vs. TM

**Figure 7:**
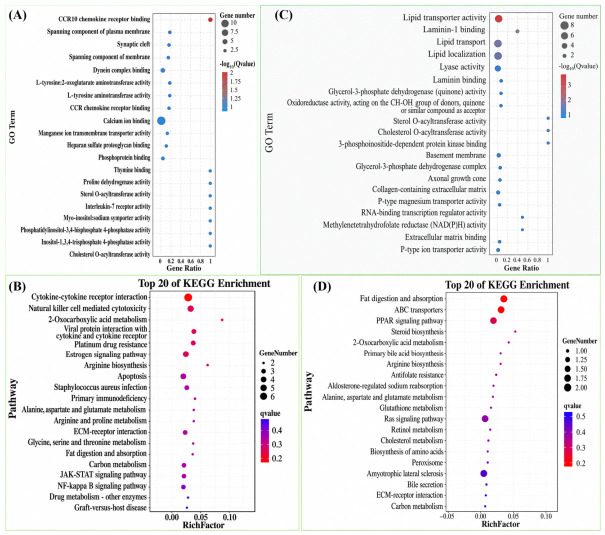
Comparative GO and KEGG enrichment profiles of NCF vs. NCM and TF vs. TM. (**A**) GO enrichment of NCF vs. NCM; (**B**) KEGG enrichment of NCF vs. NCM; (**C**) GO enrichment of TF vs. TM; (**D**) KEGG enrichment of TF vs. TM.

To further evaluate whether sex-related intestinal functional profiles differed between control and SMX-exposed conditions, we compared the functional profiles of NCF vs. NCM and TF vs. TM. GO and KEGG enrichment analyses showed that the basal sex difference in the control groups involved a broader functional spectrum than that observed between exposed females and exposed males (Figure 7). In the control groups, enriched functions were mainly related to digestion/absorption, extracellular matrix and epithelial interface-associated pathways, as well as immune- and metabolism-related signaling (Figure 7A,B; Tables S4, S11 and S12). By contrast, although TF vs. TM yielded a substantial DEG set and detectable functional differences (Table S5), the enriched functions were relatively narrower and were mainly related to metabolism- and biotransformation-associated processes (Figure 7C,D; Tables S5, S13 and S14).

GSEA analyses further identified significantly enriched functional terms and pathways that differed between NCF vs. NCM and TF vs. TM. For NCF vs. NCM, the significantly enriched GO-GSEA terms were mainly associated with RNA processing, translation, proteostasis, and protein-targeting functions, whereas the significantly enriched KEGG-GSEA pathways included digestion and absorption, extracellular matrix interaction, TGF-beta signaling, cholesterol handling, oxidative metabolism, RNA processing, and intestinal immune signaling (Tables S17 and S18). In contrast, TF vs. TM showed a more restricted set of significantly enriched GO-GSEA terms, mainly involving thyroid hormone metabolism, amino acid catabolism, aldehyde dehydrogenase-related activity, and tetrahydrofolate metabolism. The significantly enriched KEGG-GSEA pathways in TF vs. TM were mainly related to selected metabolic and biotransformation functions, including tyrosine metabolism, carbon metabolism, arginine and proline metabolism, drug metabolism-cytochrome P450, and beta-alanine metabolism (Tables S19 and S20). Together, these results showed that sex-biased functional differences were retained after SMX exposure, whereas TF vs. TM displayed a more restricted functional profile than NCF vs. NCM.

## 4. Discussion

### 4.1. Baseline Intestinal Sexual Dimorphism

The present study demonstrates that the adult intestine of *M. chulae* exhibits marked baseline sexual dimorphism at the transcriptomic level and that this dimorphism has important implications for sex-aware intestinal omics and environmental-stress evaluation in aquatic animals. At the global level, intestinal transcriptomic variation was more strongly associated with sex than with pooled treatment status (Figure 2A,B). At the differential-expression level, sex-related comparisons, including pooled F vs. M, NCF vs. NCM, and TF vs. TM, yielded substantially more DEGs than the pooled T vs. N comparison (Figure 2C; Tables S3–S8). At the pathway level, pooled F vs. M showed coordinated divergence across DEG profiles, GO/KEGG enrichment, and both GO- and KEGG-based GSEA (Figure 3, Figure 4, Figure 5 and Figure 6; Tables S3, S9, S10, S15 and S16). Together, these results indicate that the intestine in adult *M. chulae* is not a sex-neutral tissue.

This conclusion is broadly consistent with the growing recognition that sexual dimorphism extends beyond gonads and can shape intestinal biology. In fish, sex-related differences have been reported in intestinal microbial diversity, community structure, and ecological interactions, including in yellow drum and *Gymnocypris chilianensis* [12,13]. More directly, an integrated transcriptomic and metabolomic study in common carp revealed clear sex-biased intestinal molecular profiles, with female gut enriched in growth- and metabolism-related pathways and male gut enriched in immune- and disease-resistance-associated pathways [14]. Similar patterns have also been documented in mammals, where hundreds of genes show sex-biased expression in the small intestine and colon, and intestinal responses to inflammatory challenges differ between males and females [10,11]. More recent studies in teleosts have further shown that sex-specific gut immunity and microbiota dynamics can shape disease resistance under challenge conditions [16], while gut microbiota also shift substantially across sexual phenotypes during sex reversal [15]. Compared with microbiome-based studies in yellow drum, where sex affected intestinal microbial diversity, community structure, and ecological network properties [12], the present study extends the evidence for intestinal sexual dimorphism to the host transcriptomic level. The sex-related differences observed here were reflected not only in sample distribution and DEG profiles, but also in GO/KEGG enrichment and GSEA results. These findings indicate that sex-associated intestinal variation in fish can involve both microbial community organization and host gene-expression programs, highlighting the need to account for sex in intestinal omics analyses of aquatic animals.

### 4.2. Sex-Biased Intestinal Physiological Programs

A major feature of the present dataset is that female- and male-biased intestinal programs were functionally asymmetric rather than simply reciprocal. In the pooled F vs. M comparison, female-biased genes included *greb1*, *soat1*, *f13a1*, *npc1l1*, *apod*, *fabp2*, *abca1*, *fcn1*, and *vtg1* (Figure 3B; Table S3), while KEGG enrichment highlighted fat digestion and absorption, steroid biosynthesis, ABC transporters, cholesterol metabolism, and the PPAR signaling pathway (Figure 4B; Table S10). KEGG-GSEA further supported enrichment of fat digestion and absorption, cholesterol metabolism, and ECM–receptor interaction in the female-biased program (Figure 6). These results together suggest that female intestines were more strongly associated with nutrient uptake, lipid and sterol handling, and epithelial interface-related functions.

This pattern partially parallels findings in common carp, where female intestines were enriched for growth- and metabolism-related pathways, whereas male intestines showed stronger disease-resistance-associated pathways [14]. However, the specific functional axes differed between species. In common carp, the female-biased profile was mainly linked to glycolysis/gluconeogenesis and riboflavin metabolism, whereas in *M. chulae,* the female-biased program was more prominently associated with fat digestion and absorption, cholesterol metabolism, ABC transporters, and epithelial interface-related pathways. Similarly, the male-biased profile in *M. chulae* involved RNA processing, oxidative phosphorylation, and mucosal immune-related pathways, showing conceptual overlap with the immune-skewed male intestinal profile reported in common carp but differing in the specific pathways involved. Beyond fish, intestinal absorption and lymphatic transport of dietary lipids have been shown to differ between sexes in mammals, indicating that gut lipid handling can be intrinsically sex-dependent [40]. In *M. chulae*, the chromosome-level genome suggested species-specific lipid metabolic specialization, including high-fat storage-related pathways and expansion of lipid-associated genes such as ABCA1 [24]. This species background is consistent with the lipid- and sterol-related intestinal signatures observed in the present study and supports the value of *M. chulae* for investigating sex-dependent nutrient-processing traits in aquatic animal omics research.

In contrast, male-biased intestinal programs were more strongly associated with the spliceosome, oxidative phosphorylation, and the intestinal immune network for IgA production, with additional support from the ribosome and peroxisome (Figure 6 and Figure S4). Correspondingly, representative male-biased genes such as *sult1st3*, *slc5a3*, *itga1*, *casp7*, *fbxo40*, *phf6*, *nit1*, and *angptl7* suggested stronger signatures related to solute handling, RNA processing, protein turnover, oxidative metabolism, and aspects of mucosal immune signaling (Figure 3B; Table S3). The GO-GSEA results, particularly the enrichment of mRNA cis splicing via spliceosome, cytoplasmic translation, and protein targeting to the endoplasmic reticulum, further indicate that male intestines differ not only in downstream physiological output but also in the higher-order regulatory machinery governing transcript processing, translation, and intracellular trafficking (Figure 5 and Figure S3).

The structural component of the female-biased program also warrants consideration. The enrichment of ECM–receptor interaction, focal adhesion, and related epithelial interface-associated pathways suggests that the sex difference is not restricted to nutrient transport alone but may also involve epithelial organization and barrier-associated biology. This interpretation is consistent with the current understanding that intestinal barrier homeostasis is tightly linked to extracellular matrix remodeling, epithelial turnover, and mucosal immune function [41,42]. A recent fish study in juvenile turbot further showed that intestinal inflammation was accompanied by changes in ECM–receptor interaction, protein digestion and absorption, and focal adhesion, reinforcing the biological plausibility of these pathways in gut injury and barrier-associated responses [43]. Thus, the female-biased and male-biased intestinal programs identified here likely represent two distinct but biologically coherent functional configurations rather than a single continuum of sex-related expression drift. From an animal physiology perspective, these differences suggest that male and female fish intestines may differ in the molecular organization of nutrient handling, epithelial maintenance, and immune-related functions even under the same rearing and exposure conditions.

### 4.3. Sex-Aware Interpretation Under Short-Term SMX Exposure

In environmental-stress and exposure studies, baseline intestinal sexual dimorphism may reshape the interpretation of stress-induced transcriptomic responses. In the present dataset, pooled T vs. N showed no significant DEGs, whereas both NCF vs. NCM and TF vs. TM still displayed sex-related transcriptomic divergence (Figure 2C; Tables S3–S8). Moreover, the control comparison involved a broader and more structured functional spectrum than the exposed comparison (Figure 7; Tables S11–S20). Thus, short-term SMX exposure occurred on top of a pre-existing dimorphic intestinal background rather than producing a treatment-dominated transcriptomic pattern. For omics-based stress evaluation in aquatic animals, pooling females and males without distinction may therefore underestimate, dilute, or misattribute treatment-associated molecular signals, particularly when exposure effects are modest relative to intrinsic biological variation. This interpretation is consistent with recent arguments in fish immuno(eco)toxicology that biological variation, including sex, life stage, and species differences, should be explicitly considered in experimental design and data interpretation [44].

Existing studies support this concern. In fathead minnows, short-term aqueous benzo[a]pyrene exposure altered gut microbial composition in females rather than males [17]. More broadly, Bolnick et al. showed that diet–gut microbiota associations are sex-dependent, implying that sex can shape baseline gut traits as well as host responses to environmental drivers [18]. In zebrafish, although baseline intestinal microbial sex differences were not obvious, endocrine-active chemicals such as estradiol and bisphenol A altered intestinal microbiota composition [19]. More directly relevant to the present study, SMX exposure in zebrafish has been shown to induce intestinal microbiota alteration together with metabolic disturbance [20], and environmentally relevant concentrations of SMX altered the gastrointestinal microbiome of adult male and female zebrafish [21]. That study focused on microbiome-level responses, whereas the present work examined host intestinal transcriptomic patterns. Together, these findings indicate that SMX-related intestinal responses may be influenced by sex at multiple biological levels, including microbial community structure and host gene expression. More broadly, sex-based differences in contaminant toxicity have been reported across aquatic and terrestrial organisms, indicating that sex-dependent susceptibility is not restricted to a single pollutant class or biological endpoint [45].

The comparison between NCF vs. NCM and TF vs. TM further illustrates how sex-biased background variation can affect exposure–response interpretation. Under basal conditions, sex-biased intestinal divergence was functionally extensive, involving digestion/absorption, extracellular matrix and epithelial interface-associated pathways, as well as immune- and metabolism-related signaling (Figure 7A,B; Tables S4, S11 and S12). Under SMX exposure, the contrast between females and males persisted, but the functional profile became narrower and more selective, with fewer significantly enriched GSEA terms and pathways than those observed in the basal sex comparison (Figure 7C,D; Tables S5, S13, S14 and S17–S20). These results indicate that the SMX exposure response was superimposed on a pre-existing sex-biased intestinal background. Therefore, baseline sex bias provides an essential context for interpreting short-term environmental-stress responses in fish intestinal transcriptomic analyses.

### 4.4. Relevance to Omics Research in Aquatic Animals

*M. chulae* combines small body size, early maturation, high fecundity, year-round reproduction, ease of laboratory maintenance, and available genomic resources and molecular tools [23,24,25]. It has also been used in contaminant-related studies involving heavy metals, paracetamol, benzo[a]pyrene, and genotoxicity assessment [26,27,28,29,30]. These characteristics make *M. chulae* a useful marine fish model for omics-based investigations of physiological regulation, sex-related variation, and environmental stress responses. The present study adds a sex-aware transcriptomic perspective to its model utility by showing that this species can be used not only for measuring environmental stress or exposure responses, but also for evaluating biological sources of variation that influence omics-based interpretation.

This contribution is relevant to broader omics research in aquatic animals. In many omics-based transcriptomic studies of aquatic animals, sex is either pooled, incompletely reported, or treated only as a descriptive variable. The present results suggest that such practice can be problematic in intestinal studies, particularly when the exposure effect is moderate and baseline sex bias is large. Thus, *M. chulae* may be especially useful as a model for designing and testing sex-aware transcriptomic frameworks in estuarine and marine fishes. Evidence from zebrafish meta-analysis also shows that gut microbial patterns in a widely used fish model are highly structured and context-dependent [46]. Together, these observations support the broader view that model fish studies should account for biological and experimental sources of variation when interpreting intestinal responses. For the scope of aquatic animal omics, the present findings emphasize that sex-aware sampling and analysis can improve the reliability of transcriptomic interpretation in studies of intestinal physiology, health status, and environmental stress adaptation.

### 4.5. Limitations and Future Perspectives

Several limitations should nevertheless be acknowledged. First, the histological observations in this study were qualitative and were used primarily as a representative morphological context rather than as a quantitative pathology dataset (Figure 1). Quantitative morphometric indices, such as mucosal fold height, fold width, muscularis thickness or lumen area ratio, were not measured in the present study. Therefore, the histological observations were interpreted cautiously and were not used as the main evidence for sex-dependent intestinal differences. Second, the exposure design involved a single SMX concentration and a relatively short exposure period, so the current study should be interpreted as an evaluation of how sex affects transcriptomic response interpretation under one exposure scenario rather than as a full characterization of SMX-induced intestinal effects. Third, although qPCR supported the RNA-Seq trends, the validation was performed within the same experimental framework rather than in an independent biological cohort (Figure 2E; Table S1). Finally, the present study focused on transcriptomic patterns and did not directly measure circulating hormones, intestinal metabolites, microbiota composition, or physiological transport endpoints. Accordingly, the functional interpretations proposed here should be regarded as pathway-informed inferences that now warrant targeted experimental validation.

Despite these limitations, the present study provides transcriptomic evidence that intestinal sexual dimorphism in adult *M. chulae* is substantial, biologically structured, and relevant to the interpretation of environmental-stress responses. Further studies integrating hormones, metabolites, microbiota, and physiological transport endpoints will help clarify the regulatory mechanisms underlying these sex-biased intestinal programs. Future multi-omics and functional studies that explicitly include sex as an experimental factor will be important for improving the interpretation of intestinal physiology, health, and stress adaptation in aquatic animals.

## 5. Conclusions

This study characterized intestinal transcriptomic sex differences in adult *M. chulae* and evaluated their relevance to short-term SMX exposure as an environmental-stress scenario. The results showed that sex explained a greater proportion of intestinal transcriptomic variation than pooled treatment status, and sex-related comparisons produced larger DEG sets than the pooled treatment comparison. Female-biased intestinal programs were mainly associated with nutrient absorption, lipid and sterol handling, membrane transport, and epithelial interface-related pathways, whereas male-biased programs were more closely related to RNA processing, oxidative metabolism, translation, protein targeting, and mucosal immune signaling. The comparison between control and SMX-exposed sex contrasts further showed that short-term SMX exposure was superimposed on a pre-existing sex-biased intestinal background rather than producing a treatment-dominated intestinal transcriptomic profile. Overall, these findings demonstrate that the intestine of adult *M. chulae* is not a sex-neutral tissue. This study reinforces the need for sex-aware experimental design and analysis in fish intestinal transcriptomics and should encourage researchers in aquatic animal omics to consider sex an essential biological factor when evaluating intestinal physiology, health and environmental stress responses.

## Data Availability

The raw RNA sequencing data generated in this study have been deposited in the China National GeneBank DataBase (CNGBdb) under accession number CNP0009254. Other data supporting the findings of this study are available in the article and its Supplementary Materials.

## Supplementary Materials

The following supporting information can be downloaded at: https://www.mdpi.com/article/10.3390/ani16142126/s1, Figure S1: Electrophoretic quality assessment of total RNA from intestinal samples; Figure S2: Volcano plots of DEGs in pairwise comparisons; Figure S3: Additional GO-GSEA plots for the pooled female-versus-male comparison; Figure S4: Additional KEGG-GSEA plots for the pooled female-versus-male comparison; Table S1: Primer sequences used for qPCR validation; Table S2: Quality control and mapping statistics for RNA-Seq libraries; Table S3: Differentially expressed genes in the pooled female-vs-male comparison; Table S4: Differentially expressed genes in the NCF vs NCM comparison; Table S5: Differentially expressed genes in the TF vs TM comparison; Table S6: Differentially expressed genes in the NCF vs TF comparison; Table S7: Differentially expressed genes in the NCM vs TM comparison; Table S8: Gene lists for Venn diagram intersections among NCF vs NCM, TF vs TM, and pooled F vs M comparisons.; Table S9: GO enrichment results for pooled M vs F DEGs; Table S10: KEGG enrichment results for the pooled M vs F DEGs; Table S11: GO enrichment results for NCF vs NCM DEGs; Table S12: KEGG enrichment results for NCF vs NCM DEGs; Table S13: GO enrichment results for TF vs TM DEGs; Table S14: KEGG enrichment results for TF vs TM DEGs; Table S15: Full GO-GSEA results for the pooled male-vs-female comparison; Table S16: Full KEGG-GSEA results for the pooled male-vs-female comparison; Table S17: Full GO-GSEA results for NCF vs. NCM; Table S18: Full KEGG-GSEA results for NCF vs. NCM; Table S19: Full GO-GSEA results for TF vs. TM; Table S20: Full KEGG-GSEA results for TF vs. TM.
