# Supplementary material for: Comparative Intestinal Transcriptomics Reveals Sex-Dependent Physiological Signatures in Mugilogobius chulae and Supports Sex-Aware Stress Evaluation
Source: Animals (Basel). 2026 Jul 9;16(14):2126. doi: 10.3390/ani16142126 (PMC13405927; doi:10.3390/ani16142126)
Supplement: Supplementary file 1 [file animals-16-02126-s001.zip › Supplementary figures R.pdf]

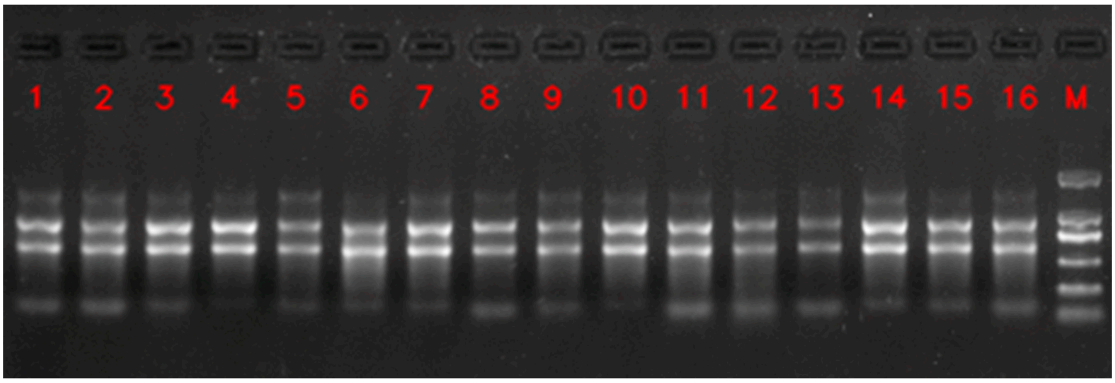

Figure S1. Electrophoretic quality assessment of total RNA from intestinal samples.

Note: Lanes 1–4: control females; 5–8: control males; 9–12: SMX-exposed females; 13–16: SMX-exposed males; M: Marker

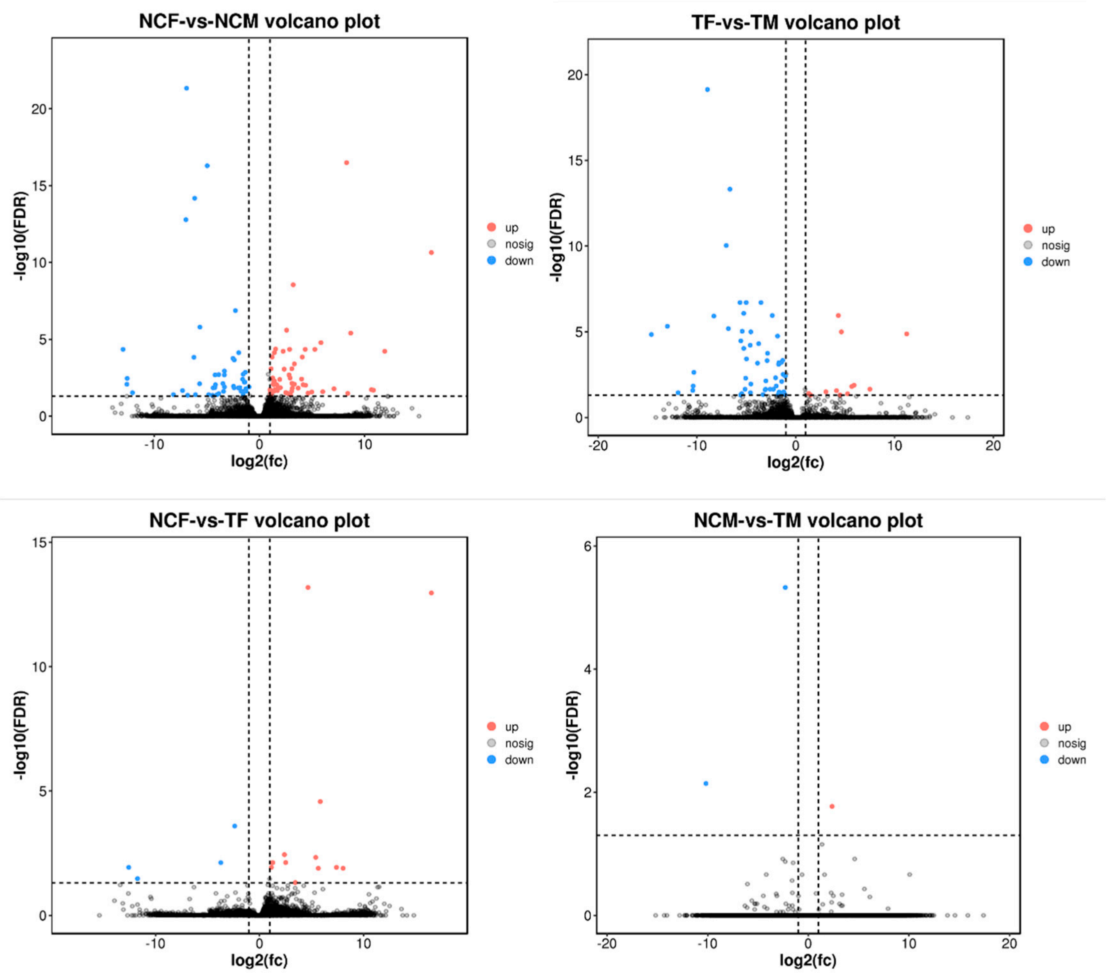

Figure S2. Volcano plots of DEGs in pairwise comparisons. (A) NCF vs NCM; (B) TF vs TM; (C) NCF vs TF; (D) NCM vs TM.

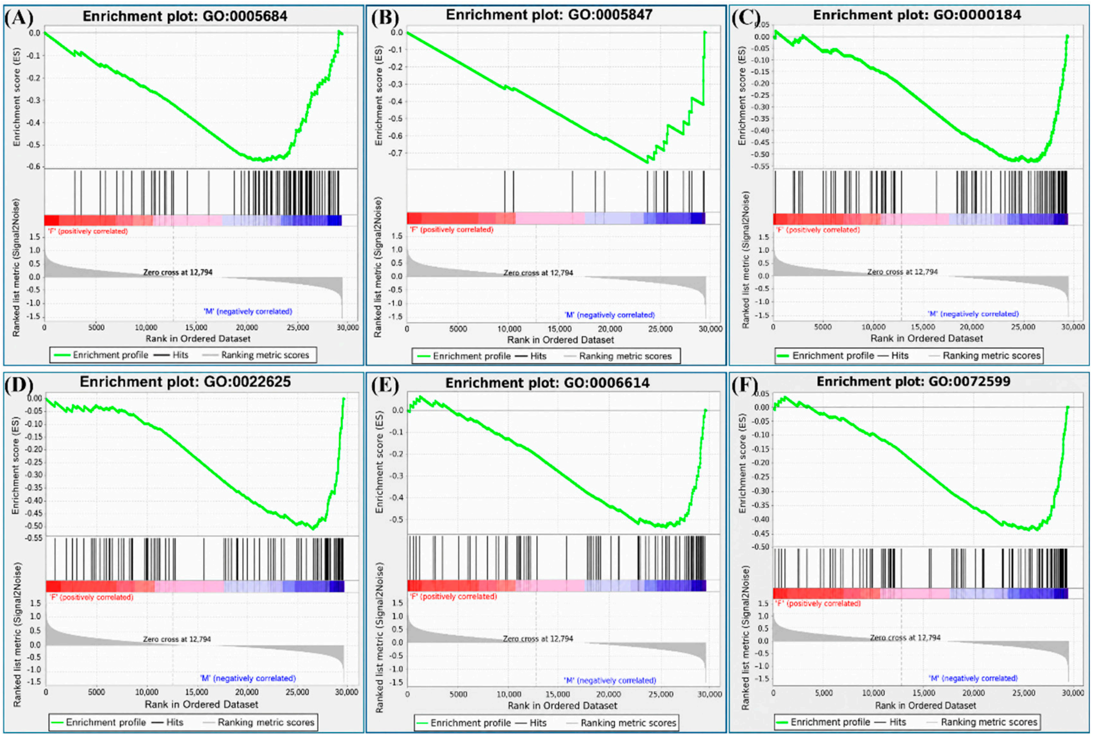

Figure S3. Additional GO-GSEA plots for the pooled female-versus-male comparison. (A) U2-type spliceosomal complex; (B) mRNA cleavage and polyadenylation specificity factor complex; (C) nuclear-transcribed mRNA catabolic process, nonsense-mediated decay; (D) cytosolic large ribosomal subunit; (E) SRP-dependent cotranslational protein targeting to membrane; (F) establishment of protein localization to endoplasmic reticulum.

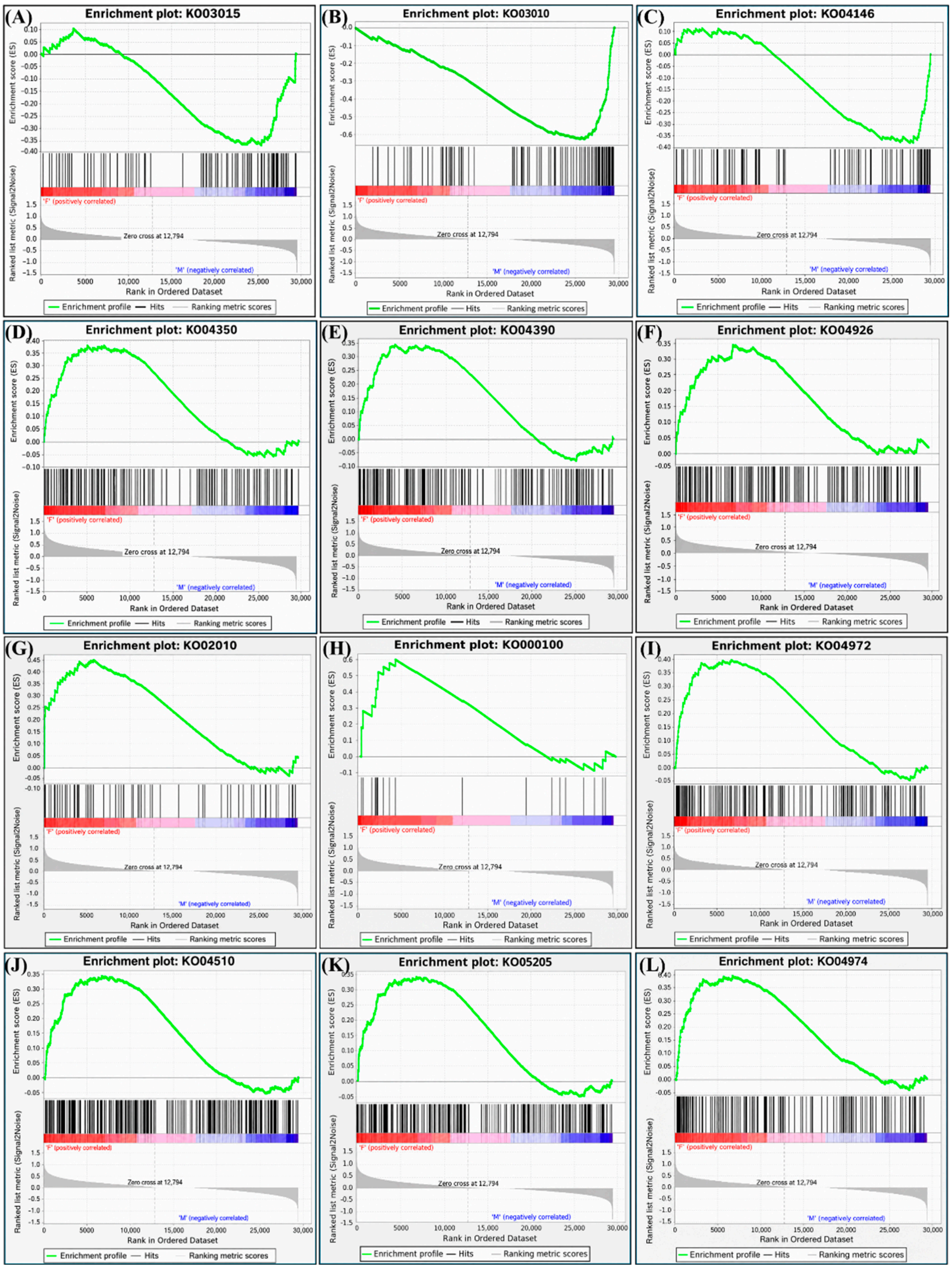

Figure S4. Additional KEGG-GSEA plots for the pooled female-versus-male comparison. (A) mRNA surveillance pathway; (B) ribosome; (C) peroxisome; (D) TGF-beta signaling pathway; (E) Hippo signaling pathway; (F) relaxin signaling pathway; (G) ABC transporters; (H) steroid biosynthesis; (I) pancreatic secretion; (J) focal adhesion; (K) proteoglycans in cancer; (L) protein digestion and absorption.
