# Supplementary material for: Comparative Intestinal Transcriptomics Reveals Sex-Dependent Physiological Signatures in Mugilogobius chulae and Supports Sex-Aware Stress Evaluation
Source: Animals (Basel). 2026 Jul 9;16(14):2126. doi: 10.3390/ani16142126 (PMC13405927; doi:10.3390/ani16142126)
Supplement: Supplementary file 1 [file animals-16-02126-s001.zip › Table S1.pdf]

Table S1. Primer sequences used for qPCR validation.

Gene symbol	Primer sequence (5′–3′)	Amplicon size (bp)
ty3b-g	**F:** TAGCGACGAGGACTATGGACTG; **R:** ATTTGTGGTGGTATTTGGCG	102
greb1l	**F:** CGCAGCCTCTTATCTCAAGTCTG; **R:** TCCACAAGCACACCTGATTG	161
lrrc58	**F:** CAGTGTCAGCGACGAGAGAAAG; **R:** TGTCCAGAAACTCCAGGTTGC	113
tuba1a	**F:** TCTGGAGCCAACTGTCATAGATG; **R:** CTTTGCCGATGGTGTAGTGTC	131
ppef2	**F:** GATGGTATCGTCAGTATGTTGCG; **R:** GTTCGTTGCTTGATGGAGTG	156
retsatl	**F:** GGATGTGATTGTGATTGGCAG; **R:** CCCTTCTCTATGTATGTGTGACAGC	135
f13a1	**F:** TGGAGTTCAGTGGTTGGCAG; **R:** TGTTCACCTCAGCGAAGACG	150
greb1	**F:** GTGGTTCTGACTGCGGTTTG; **R:** ATTGTAAGGGCTGATTCGGG	143
fabp2	**F:** GGAAAGTTCACCAGGAAAGACAAC; **R:** TGGCGTCCACACCATCATAG	117
dpys	**F:** CATTGATGCCACTGACAAACTG; **R:** TCTGAGGGATGACAAAGTCCAG	164
casp6	**F:** AGATGGAGGGTATTCTCAGTGAAG; **R:** GCAGTGATGTCGTTGATGTTG	139
casp7	**F:** TGAGGACAAATACCCGATGG; **R:** AAAGTCTTCACCAGAGCGTCTC	135
MSTRG.20163	**F:** TGTGTCCCTGAGAATGCTCC; **R:** CGCCAGTCTGCCTTCAATAG	120
MSTRG.25197	**F:** GAGCGAGAGCCAGACCATAAG; **R:** GAGCACCAGCACTAACACAAGTC	94
dnah7	**F:** TGTTCTTCTCCATCGCCGAC; **R:** TCGCTCTTCTCCGAGTTGTC	109
soat1	**F:** GCTCAAGCATTTATGGACGC; **R:** TTAGGGATGGCACGACAGC	145
β-actin	**F:** GGCTACTCCTTCACCACCACAG; **R:** TTCCGCAAGATTCCATACCG	232
gapdh	**F:** CGCATCGGTCGTCTGGT; **R:** GGGTGGAGTCATACTTGAACAT	121

note: *mstrg id*s represent transcript identifiers generated from RNA-seq assembly.
