# Supplementary material for: Comparative Intestinal Transcriptomics Reveals Sex-Dependent Physiological Signatures in Mugilogobius chulae and Supports Sex-Aware Stress Evaluation
Source: Animals (Basel). 2026 Jul 9;16(14):2126. doi: 10.3390/ani16142126 (PMC13405927; doi:10.3390/ani16142126)
Supplement: Supplementary file 1 [file animals-16-02126-s001.zip › Table S2.pdf]

**Table S2. Quality control and mapping statistics for RNA-Seq libraries.**

| Sample | Raw Data(bp) | Clean Data(bp) | Q20 bases (%) | Q30 bases (%) | GC bases (%) | Unmapped reads (%) | Unique Mapped reads (%) | Multiple Mapped reads (%) | Total Mapped reads (%) |
|---|---|---|---|---|---|---|---|---|---|
| NCF_1 | 5589270600 | 5513003463 | 5375536584 (97.51%) | 5139041705 (93.22%) | 2642932958 (47.94%) | 6525168 (17.58%) | 29352126 (79.09%) | 1234756 (3.33%) | 30586882 (82.42%) |
| NCF_2 | 5510174400 | 5439710742 | 5307061719 (97.56%) | 5077856148 (93.35%) | 2587426533 (47.57%) | 6307308 (17.24%) | 29201595 (79.80%) | 1085395 (2.97%) | 30286990 (82.76%) |
| NCF_3 | 6306570000 | 6224550230 | 6058233235 (97.33%) | 5780103955 (92.86%) | 2974243858 (47.78%) | 7614229 (18.19%) | 32992810 (78.81%) | 1256819 (3.00%) | 34249629 (81.81%) |
| NCF_4 | 5953526700 | 5882700878 | 5718189553 (97.20%) | 5445594828 (92.57%) | 2812897335 (47.82%) | 7590713 (19.21%) | 30756904 (77.82%) | 1174651 (2.97%) | 31931555 (80.79%) |
| NC_M1 | 5668709700 | 5591013398 | 5451935858 (97.51%) | 5211340725 (93.21%) | 2597476399 (46.46%) | 8104447 (21.54%) | 28551725 (75.87%) | 977560 (2.60%) | 29529285 (78.46%) |
| NC_M2 | 6606891300 | 6529500304 | 6385983075 (97.80%) | 6128733422 (93.86%) | 3103025820 (47.52%) | 7840088 (17.87%) | 34688458 (79.07%) | 1341532 (3.06%) | 36029990 (82.13%) |
| NC_M3 | 6344860200 | 6265555888 | 6144245768 (98.06%) | 5909215780 (94.31%) | 2991958429 (47.75%) | 7837025 (18.60%) | 32972373 (78.25%) | 1329044 (3.15%) | 34301417 (81.40%) |
| NC_M4 | 6598268100 | 6527579242 | 6374249886 (97.65%) | 6106528563 (93.55%) | 3052338973 (46.76%) | 8878441 (20.28%) | 33660239 (76.87%) | 1250648 (2.86%) | 34910887 (79.72%) |
| TF_1 | 5715945000 | 5632594828 | 5499133416 (97.63%) | 5265280986 (93.48%) | 2617888443 (46.48%) | 7327930 (19.30%) | 29511750 (77.74%) | 1120582 (2.95%) | 30632332 (80.70%) |
| TF_2 | 6071997300 | 6009979267 | 5862852312 (97.55%) | 5603130480 (93.23%) | 2872309374 (47.79%) | 7092705 (17.59%) | 31897775 (79.11%) | 1328380 (3.29%) | 33226155 (82.41%) |
| TF_3 | 6742750800 | 6484320590 | 6338301562 (97.75%) | 6078826108 (93.75%) | 3083630692 (47.56%) | 7911789 (17.66%) | 35534952 (79.33%) | 1345561 (3.00%) | 36880513 (82.34%) |
| TF_4 | 6647646600 | 6597076868 | 6450964158 (97.79%) | 6188124403 (93.80%) | 3095681600 (46.93%) | 8934660 (20.23%) | 33948278 (76.88%) | 1272472 (2.88%) | 35220750 (79.77%) |
| TM_1 | 5611290300 | 5547586692 | 5404116740 (97.41%) | 5161750382 (93.04%) | 2609412283 (47.04%) | 7216121 (19.38%) | 28996259 (77.86%) | 1029630 (2.76%) | 30025889 (80.62%) |
| TM_2 | 6482911800 | 6413578536 | 6251821893 (97.48%) | 5974714721 (93.16%) | 3087489410 (48.14%) | 7149072 (16.62%) | 34562561 (80.34%) | 1310507 (3.05%) | 35873068 (83.38%) |
| TM_3 | 5865261300 | 5811450203 | 5662288950 (97.43%) | 5408957289 (93.07%) | 2716049916 (46.74%) | 7929039 (20.35%) | 29931250 (76.81%) | 1106119 (2.84%) | 31037369 (79.65%) |
| TM_4 | 6123726900 | 6075449461 | 5932456155 (97.65%) | 5676405718 (93.43%) | 2926118227 (48.16%) | 6900021 (16.97%) | 32543250 (80.03%) | 1221561 (3.00%) | 33764811 (83.03%) |
